# Ginseng-based carbon dots inhibit the growth of squamous cancer cells by increasing ferroptosis

**DOI:** 10.3389/fonc.2023.1097692

**Published:** 2023-03-10

**Authors:** Zilin Wang, Jing Han, Zhiyong Guo, Hao Wu, Yige Liu, Wenying Wang, Chenping Zhang, Jiannan Liu

**Affiliations:** ^1^ Department of Oral Maxillofacial - Head & Neck Oncology, Shanghai Ninth People’s Hospital Affiliated to Shanghai Jiao Tong University School of Medicine, National Clinical Research Center for Oral Diseases, Shanghai Key Laboratory of Stomatology, Shanghai Research Institute of Stomatology, Shanghai, China; ^2^ College of Stomatology, Shanghai Jiao Tong University, Shanghai, China; ^3^ Shanghai Key Laboratory of Tissue Engineering Research, Shanghai, China; ^4^ School of Health Science and Engineering, University of Shanghai for Science and Technology, Shanghai, China

**Keywords:** carbon dots, ginseng, nanomaterials, ferroptosis, squamous cancer, neoplasm invasiveness

## Abstract

**Background:**

Recent studies indicated that Ginseng potentiate cancer treatments. Ginseng-based carbon dots (GCDs) might possess properties to kill cancer cells and inhibit malignant tumor development and invasion. This study aimed to prepare GCDs, examine their effects on cancer cell growth and invasion, and explore the mechanisms involved.

**Methods:**

GCDs were synthesized, purified, and characterized. Cells were cultured with GCDs and were tested for growth, invasiveness, and wound healing. RNA was extracted for transcriptomics analysis. Protein expression was evaluated using western blot and immunohistochemistry. Mice were injected with cancer cells and treated with PBS or GCDs. Tumor volume was evaluated.

**Results:**

GCDs were successfully synthesized and purified. The solution was yellow under sunlight and fluorescent blue under ultraviolet light. Electron microscopy showed GCDs with a uniform shape without apparent aggregation and an average diameter of about 4 nm. GCDs inhibited Cal-27, SCC-25, and SCC-7 cancer cell growth at concentrations of >250-300 μg/mL, while GCDs inhibited the non-cancerous HaCaT cells at concentrations >400 μg/mL. Immunofluorescence showed that GCDs could enter the cells. Transcriptomics revealed 552 downregulated mRNAs and 338 upregulated ones, including mRNAs involved in the oxidative phosphorylation and ferroptosis pathways. GCDs induced the ferroptosis of cancer cells, as shown by decreased GPX-4 and increased COX-2. GCDs decreased cell invasion and migration. *In vivo*, GCDs decreased tumor growth without apparent organ toxicity and promoted CD4^+^ T cell infiltration in the tumor.

**Conclusion:**

GCDs appear to possess anticancer properties by increasing ferroptosis, resulting in cancer cell growth inhibition *in vitro* and *in vivo*.

## Introduction

There were an estimated 19.3 million new cancer cases and 10.0 million cancer-related deaths in 2020 (1). The burden of cancer has increased in recent years, owing to population aging and socioeconomic development, since many risk factors for cancer are associated with a higher socioeconomic status (1–3). Improving cancer prevention and control to improve public health outcomes (4). Despite ever-improving treatments and new drugs (5, 6), cancer still takes its toll (1, 7).

Cancer-related death is usually due to the failure of the primary organ due to cancer growth or metastasis that causes distant organ failure (8, 9). In both cases, cancer cell growth and invasion are involved (10, 11). Currently, radiotherapy, chemotherapy, and targeted therapies are routine treatments to prevent recurrence and metastasis by killing cancer cells locally or distantly (5, 12, 13). Still, these approaches have several shortcomings, including toxicity, reduced quality of life, risk of secondary cancer, and cancer resistance (5, 14, 15). In addition, even though attacking cancer from multiple fronts is an appealing concept, doing so is often impossible because of the combined toxicities of the different cancer drugs, and such strategies must be used in highly selected patients (16, 17).

Therefore, traditional herbal medicine could provide add-on treatments that would not increase the toxicity of conventional cancer treatments, and that could also be used over the long term after the completion of standard cancer treatment regimens (18, 19). Ginseng is a traditional herbal medicine that has been used for a long time in Asia (20, 21), including for cancer (22, 23). Recent studies indicated that Ginseng could potentiate cancer treatments (24). Still, the functional composition of Ginseng is unclear, and the route of administration is inadequate to meet the needs of tumor treatment.

Nano-sized traditional herbal medicines have good application prospects, especially in preparing the herbal medicines into carbon dots, which can effectively improve their therapeutic effect. Carbon dots are less than 10 nm in diameter, and they can enter the cells easily, cross the blood-brain barrier, and perform biological functions (25, 26). Ginseng-derived nanoparticles could have promising prospects in cancer treatment (27, 28). Ginseng-based carbon dots (GCDs) might possess properties to kill cancer cells and inhibit malignant tumor development and invasion.

Hence, the present study aimed to prepare GCDs, examine their effects on cancer cell growth and invasion, and explore the mechanisms involved. GCDs might provide a new method and theoretical basis for saving patients’ lives.

## Materials and methods

Synthesis and purification of GCDs, characterization of the GCDs, cell culture, ROS detection, cytotoxicity assays, western blot, Transwell assay, wound healing assay, animal experiments, and statistical analysis are described in the **Supplementary Materials**.

### Construction of RNA sequencing libraries and sequencing

Total RNA was extracted from the samples by Trizol reagent (Invitrogen Inc., Carlsbad, CA, USA). The RNA quality was checked using an Agilent 2200 system (Agilent Technologies, Santa Clara, CA, USA). RNA was kept at -80°C. The RNA with RIN (RNA integrity number) >7 was acceptable for cDNA library construction. The cDNA libraries were constructed for each RNA sample using the TruSeq Stranded mRNA Library Prep Kit (Illumina, Inc., San Diego, CA, USA) according to the manufacturer’s instructions. Poly-A containing mRNA was purified from 1 µg of total RNA using oligo(dT) magnetic beads and fragmented into 200-500 bp using divalent cations at 85°C for 6 min. The cleaved RNA fragments were used for first- and second-strand complementary DNA (cDNA) synthesis. dUTP mix was used for second-strand cDNA synthesis, which allows for the removal of the second strand. The cDNA fragments were end-repaired, A-tailed, and ligated with indexed adapters. The ligated cDNA products were purified and treated with uracil DNA glycosylase to remove the second-strand cDNA. The purified first-strand cDNA was enriched by PCR to create the cDNA libraries. The libraries were quality controlled with an Agilent 2200 and sequenced by HiSeq X (Illumina) on a 150 bp paired-end run.

### RNA sequencing mapping

Before reading, clean reads were obtained from the raw reads by removing the adaptor sequences and low-quality ones. The clean reads were aligned to the human genome (GRCh38, NCBI) using Hisat2 (29). HTseq (30) was used to get gene counts. The RPKM method was used to determine the gene expression.

### Dif-gene analysis

The EB-Seq algorithm (31) was used to filter the differentially expressed genes. Then, FDR analysis (32) was performed under the following criteria: i) fold change >2 or <0.5, and ii) FDR <0.05.

### GO analysis

Gene ontology (GO) analysis was performed to elucidate the biological implications of the differentially expressed genes in the experiment (33). The GO annotations were downloaded from NCBI (http://www.ncbi.nlm.nih.gov/), UniProt (http://www.uniprot.org/), and the Gene Ontology (http://www.geneontology.org/). Fisher’s exact test was applied to identify the significant GO categories (P-value <0.05).

### Pathway analysis

Pathway analysis was used to find out the significant pathway of the differentially expressed genes according to the KEGG database. The Fisher’s exact test was used to select the significant pathways, and the threshold of significance was defined by P-value <0.05 (34).

## Results

### Synthesis and characterization of the GCDs

**Figure 1:**
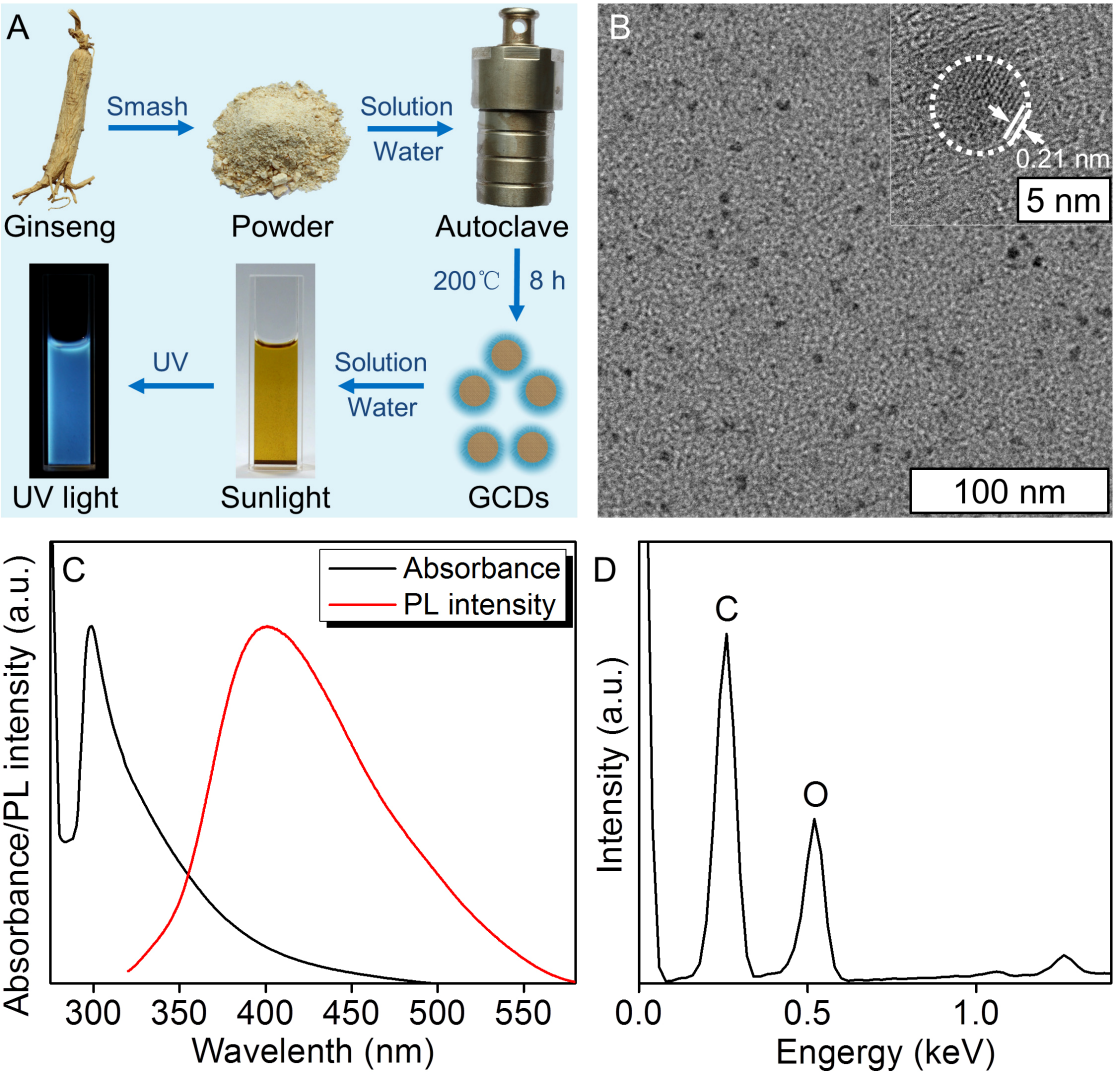
Synthesis and characterization of ginseng-based carbon dots (GCDs). **(A)** Synthesis of GCDs and the sunlight and UV light images of the GCD solution. **(B)** High-resolution transmission electron microscope (HRTEM) image of GCDs with a diameter of around 4 nm and a lattice structure of 0.21 nm. **(C)** Photoluminescent (PL) emission and UV-vis spectroscopy of absorption of the GCD solution. The PL emission peaked at 400 nm, and the UV-vis spectroscopy of the absorbance peak was at 298 nm. **(D)** Energy dispersive spectrum (EDS) of GCDs.

In this study, the GCDs were prepared from Ginseng in Northeast China by the solvothermal procedure (35). During this process, the mash of the Ginseng was added to the solvothermal autoclave reactor, and the heat of carbonization led to the formation of GCDs. The solution was yellow under sunlight and fluorescent blue under ultraviolet light (**Figure 1A**). In order to define the properties of the GCDs, a high-resolution transmission electron microscope (HRTEM) image was obtained (**Figure 1B**), showing GCDs with a uniform shape without apparent aggregation and an average diameter of about 4 nm and a lattice structure of 0.21 nm. The nanosize of GCDs allows them possible to get into the cells and perform their pharmacological functions. The as-prepared GCDs solution exhibited a strong photoluminescent (PL) emission, with the PL emission peak at 400 nm and the UV-vis spectroscopy of the absorbance peak of GCDs at 298 nm (**Figure 1C**). The energy dispersion spectrum (EDS) demonstrated that GCDs are composed of C and O (**Figure 1D**). All the results demonstrate that the prepared GCDs possess nanometric size and fluorescence and can be used for further biological applications.

### GCDs inhibit cancer cell growth and can enter the cells

**Figure 2:**
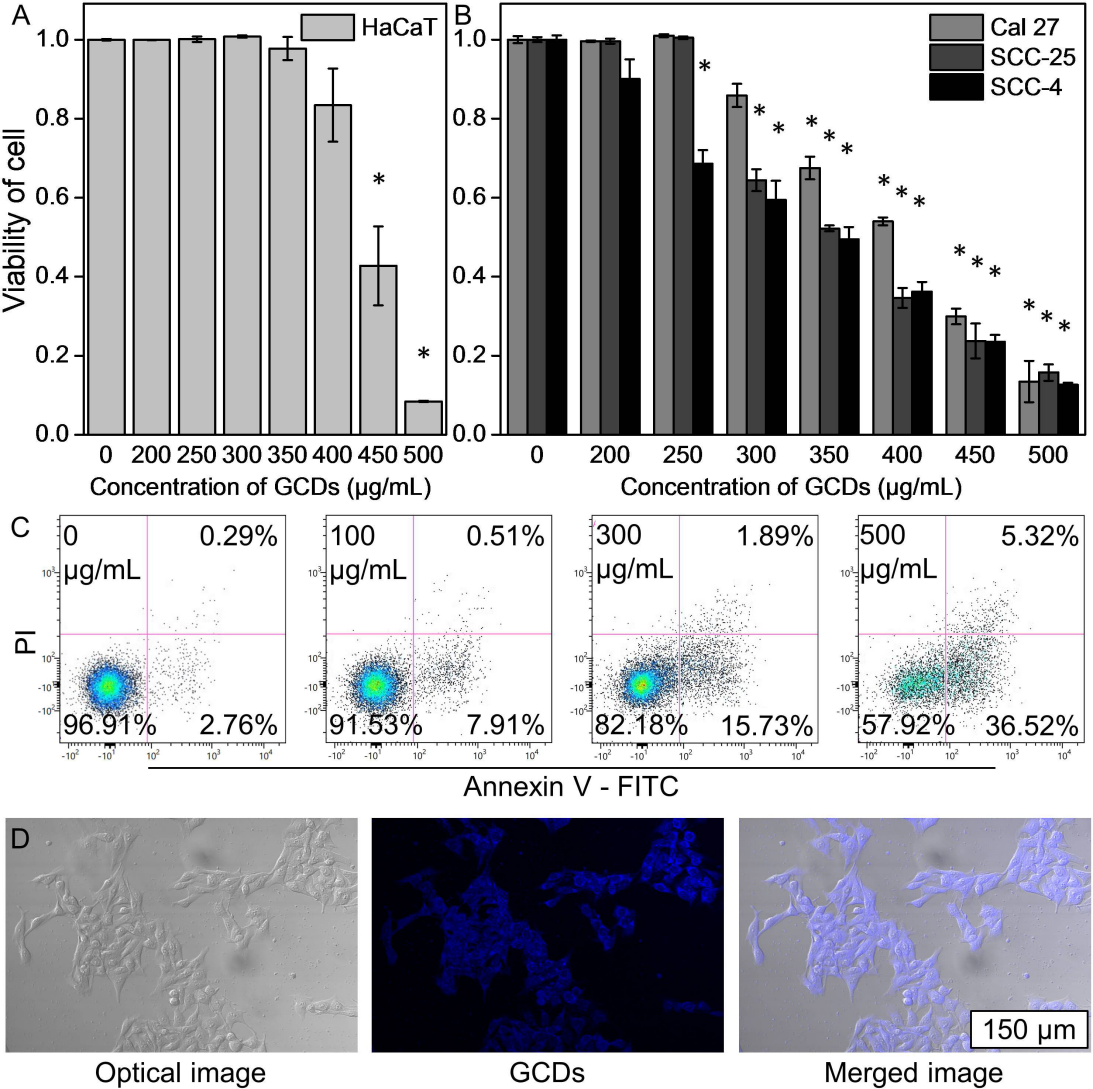
Biocompatibility and cell imaging of ginseng-based carbon dots (GCDs). **(A)** Cell counting kit-8 (CCK-8) assay of normal immortalized keratinocytes (HaCaT cells) treated with GCDs with different concentrations from 0 to 500 μg/mL. **(B)** CCK-8 assay of squamous cell carcinoma cell lines (Cal-27, SCC-25, and SCC-7) treated with GCDs with different concentrations from 0 to 500 μg/mL. **(C)** Apoptosis assay by flow cytometry in Cal-27 cells treated with different concentrations of GCDs from 0 to 500 μg/mL. **(D)** Cal-27 cells were treated by GCDs (300 μg/mL) by fluorescence microscopy. Data are presented as mean ± SD from three experiments. * P<0.05.

The biocompatibility of the GCDs was evaluated by the CCK-8 assay to determine whether GCDs can be used for further studies *in vivo* and *in vitro*. The CCK-8 assay showed that GCDs have a growth inhibition effect that could be seen at higher concentrations (>400 μg/mL) in normal immortalized keratinocytes cell line (HaCaT) (**Figure 2A**). GCDs had no significant effects on cell proliferation in squamous cell carcinoma cell lines (Cal-27, SCC-25, and SCC-7) at concentrations >250-300 μg/mL, but GCDs have a growth inhibition effect that could be seen at higher concentrations in Cal-27, SCC-25, and SCC-7 cells (**Figure 2B**). The results indicate that GCDs might have the specificity of killing cancer cells instead of normal cells. Apoptosis was analyzed using flow cytometry. The apoptosis assay of Cal-27 cells (squamous cell carcinoma) treated with GCDs at different concentrations showed that GCDs could induce apoptosis of Cal-27 cells at concentrations from 300 μg/mL (**Figure 2C**). Live/Dead staining also show GCD induce Cal-27 cell death (**Figure S3**) Those results demonstrate that GCDs have good biocompatibility for normal cells and are specific to killing tumor cells. Furthermore, Cal-27 cells were incubated with GCDs at 300 μg/mL for 24 h, and the image of fluorescence microscopy was taken. The images show blue fluorescence around and within the cells, indicating that GCDs could enter the cells (**Figure 2D**).

### GCDs affect cancer cell transcriptomics

**Figure 3:**
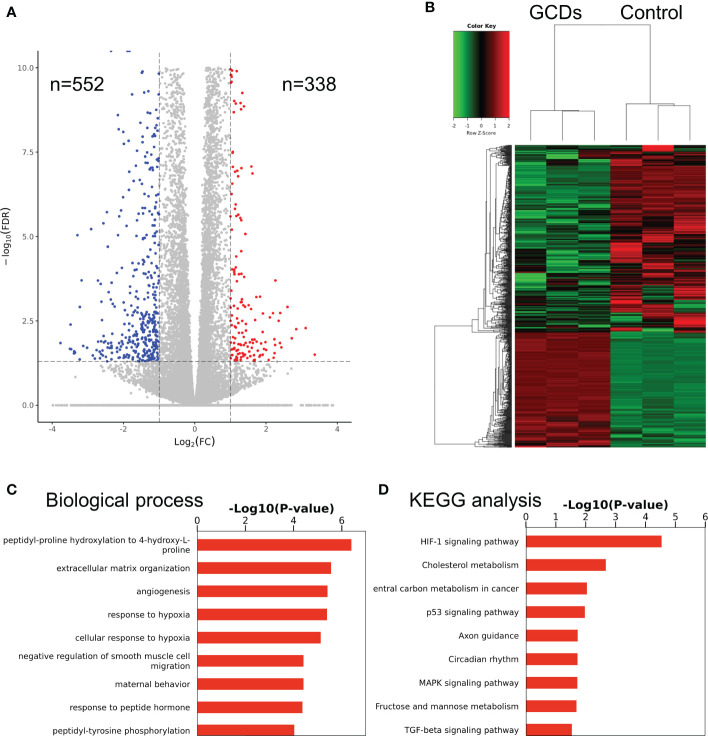
RNA sequencing of cells treated with ginseng-based carbon dots (GCDs). **(A)** Volcano map for the changes in gene expression in Cal-27 cells treated with GCDs. **(B)** Heatmap for the changes in gene expression in Cal-27 cells treated with GCDs. **(C, D)** GO and KEGG analysis for all genes with altered expressions in Cal-27 cells treated with GCDs.

In order to confirm the differences between the GCD and control groups, the RNA-seq analysis was applied to determine the differentially expressed mRNAs for Cal-27 cells. The results showed significant changes after GCD induction, with 552 downregulated mRNAs and 338 upregulated ones (**Figures 3A, B**, **S6A, B**). **Figures 3C** and S6a show that the most enriched biological processes were peptidyl-proline hydroxylation, extracellular matrix organization, angiogenesis, response to hypoxia, cellular response to hypoxia, negative regulation of smooth muscle cell migration, maternal behavior, response to peptide hormone, and peptidyl-tyrosine phosphorylation. **Figures 3D** and **S6B** show that the most enriched KEGG pathways were HIF-1 signaling, cholesterol metabolism, carbon metabolism in cancer, p53 signaling, axon guidance, circadian rhythm, MAPK signaling, fructose and mannose metabolism, and TGF-β signaling.

### GCDs-induces the ferroptosis of cancer cells

**Figure 4:**
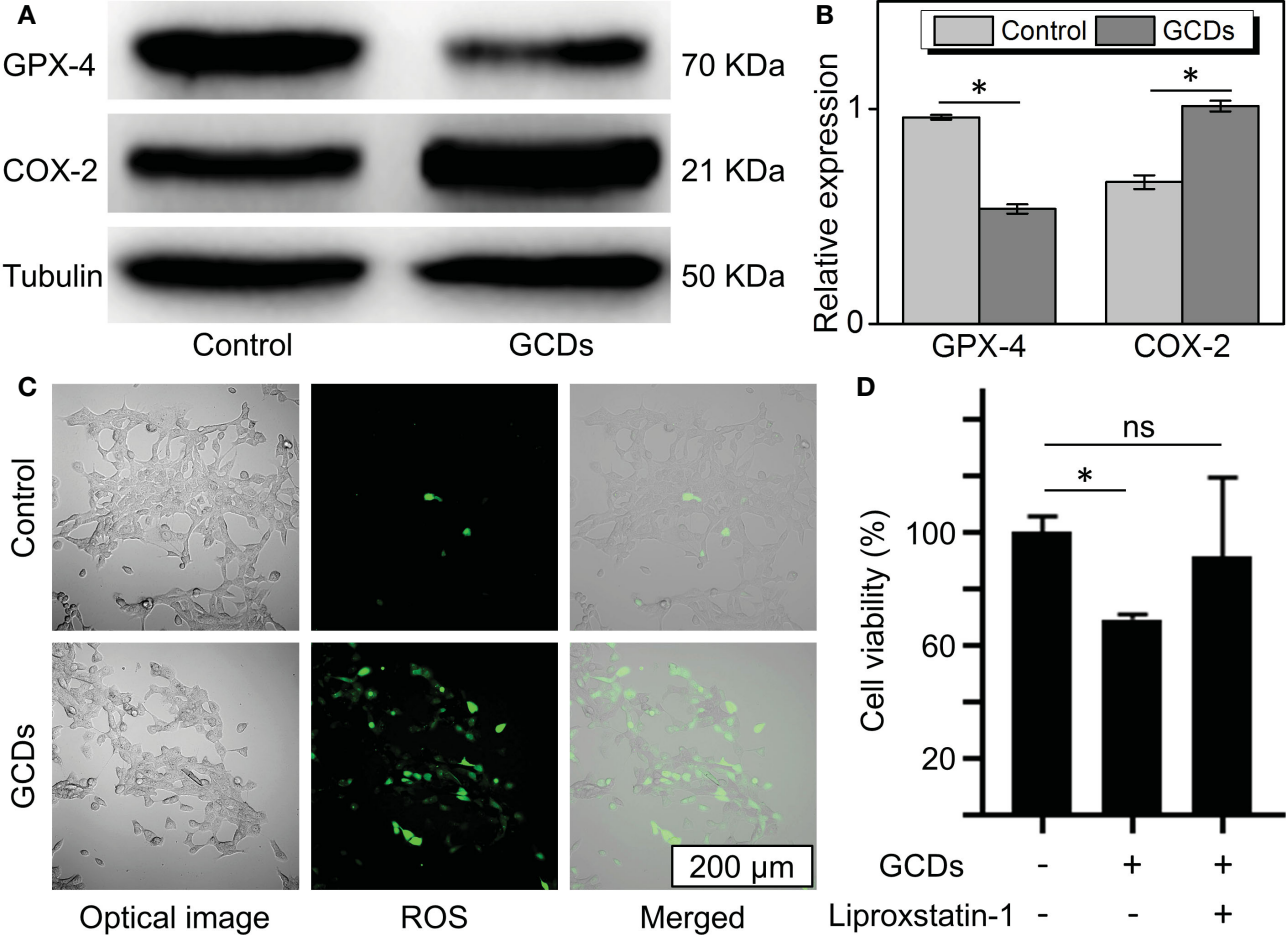
Ginseng-based carbon dots (GCDs)-induced ferroptosis of cancer cells. **(A, B)** Western blot of ferroptosis-related protein (GPX-4 and COX-2) expression of Cal-27 treated with GCDs (300 μg/mL) for 24 h. **(C)** Images of reactive oxygen species (ROS) expression assay with green fluorescence. **(D)** Cell counting kit-8 (CCK-8) assay for the viability of cancer cells treated with GCDs or the inhibitor of ferroptosis (liproxstatin-1). Data are presented as mean ± SD from three experiments. * P<0.05; ns stands for Not Statistically.

From the RNA sequencing results, GCDs were found to affect the oxidative phosphorylation and ferroptosis pathways. Therefore, the death of the tumor cells induced by GCDs could be related to ferroptosis. In order to demonstrate the relationship between GCDs and tumor cell death, western blot was carried out to detect the ferroptosis-related protein expression. The results show that glutathione peroxidase 4 (GPX-4) was decreased, and cyclooxygenase-2 (COX-2) was increased in Cal-27 incubated with GCDs at 300 μg/mL (**Figures 4A, B**). The intracellular reactive oxygen species (ROS) assay was used to detect the ROS expression in tumor cells treated with GCDs. ROS can oxidize the non-fluorescent 2’,7’-dichlorofluorescein diacetate (DCFH-DA) probe and into green fluorescent 2’,7’-dichlorofluorescein (DCF). The images show green fluorescence (ROS) in Cal-27 cells incubated with GCDs, indicating high ROS levels with 27 times increase (**Figure S4**), whereas the control group showed low ROS levels (**Figure 4C**). Flow cytometry analysis reveals a significant increase in ROS expression in Cal-27 cells after GCD treatment (**Figure S5**). The inhibitor of ferroptosis, liproxstatin-1, was used to test the effect of GCDs on ferroptosis. The CCK-8 assay showed that the viability of Cal-27 cells incubated with both GCDs and liproxstatin-1 returned to normal, demonstrating that GCDs can induce ferroptosis to kill the tumor cells (**Figure 4D**).

### GCDs inhibit tumor invasion and migration

**Figure 5:**
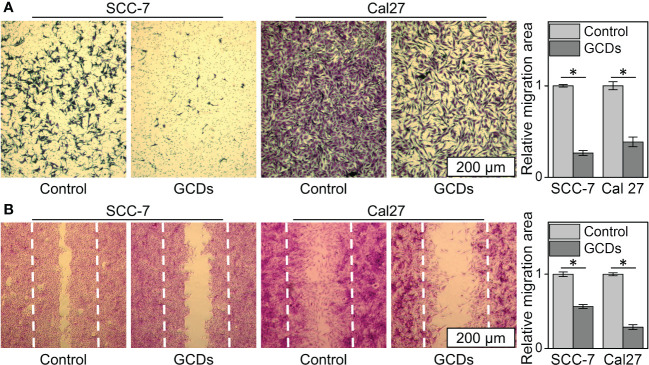
Ginseng-based carbon dots (GCDs) inhibit tumor invasion and migration. **(A)** Transwell assay of SCC-7 and Cal27 cells treated with GCDs (300 μg/mL). **(B)** Wound healing assay of SCC-7 and Cal27 cells treated with GCDs (300 μg/mL). Data are presented as mean ± SD from three experiments. * P<0.05.

The wound healing and Transwell assays were carried out to investigate the changes in cell motility. The Transwell assay showed that the percentages of GCD-induced Cal-27 and SCC-7 cells that migrated to the inferior membrane were approximately 20% and 30% compared to the control group, respectively (**Figure 5A**). In the wound healing assay, as seen in **Figure 5B**, Cal-27 cells and SCC-7 cells without GCDs treatment exhibit a higher cell migration than those co-cultured with GCDs.

### GCDs inhibit tumor growth *in vivo*

**Figure 6:**
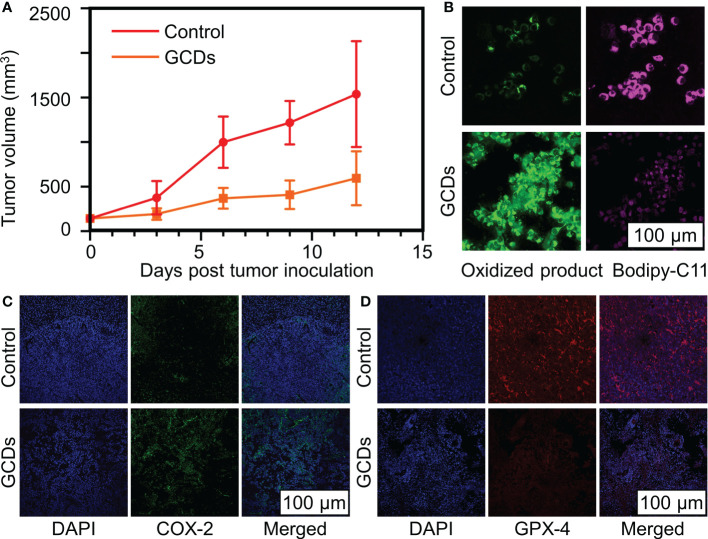
Ginseng-based carbon dots (GCDs) inhibit cancer growth *in vivo*. **(A)** Tumor volume (mm^3^) on mice after being treated with GCDs compared to the control group over 12 days. **(B)** Reactive oxygen species (ROS) levels in cancer cells by immunofluorescence for bodyipy-C11 sensitivity. **(C, D)** Immunofluorescence of tumor for COX-2 and d GPX-4 expression in control and GCDs group. Data are presented as mean ± SD from three experiments.

In order to confirm the GCDs’ inhibition of cancer growth, the tumor volume (mm^3^) was evaluated in xenograft mouse models. **Figure 6A** shows that the tumor volume grew more rapidly in control animals than in GCD-treated ones. **Supplementary Figure S1** shows no differences in heart, liver, spleen, and kidney histology between the two groups, suggesting no obvious toxicity in mice. Tumor lipid peroxidation was detected by the fluorescent bodipy-C11 probe, in which ROS expression will shift the bodipy-C11 probe from magenta to green. **Figure 6B** shows increased lipid oxidation in GCD-treated tumors. **Figures 6C, D** shows increased COX-2 and decreased GPX-4 in the GCD-treated animals, consistent with the *in-vitro* experiments. **Supplementary Figure S2** shows higher numbers of CD4^+^ T cells and lower numbers of regulatory T cells in GCD-treated tumors.

## Discussion

This study aimed to prepare GCDs, examine their effects on cancer cell growth and invasion, and explore the mechanisms involved. The results suggest that GCDs appear to possess anticancer properties by increasing ferroptosis, resulting in cancer cell growth inhibition *in vitro* and *in vivo*. The inhibitory dose was lower in cancer cells than in normal cells. *In vivo*, GCDs decreased tumor growth without apparent organ toxicity and promoted CD4^+^ T cell infiltration in the tumor.

Ginseng is a traditional herbal medicine against cancer (22, 23). Therefore, ginseng-derived nanoparticles could have promising prospects in cancer treatment (27, 28). Indeed, ginseng preparations have inhibited melanoma cells (27). Ginseng, in combination with conventional cancer treatments, has shown promising prospects in various cancer types (36). Carbon dots are promising drug vehicles since they can easily enter the cells and even cross the blood-brain barrier (25, 26). Carbon dots can easily carry active ginseng compounds in the cells. The use of GCD has high biocompatibility and can effectively avoid the body’s resistance to external substances. The present study showed that GCDs could inhibit cancer cells using a lower concentration than the inhibitory concentration in normal cells. In addition, GCDs slowed the tumor growth in mice without affecting the histology of the heart, liver, spleen, and kidney. These characteristics are important for the eventual use of Ginseng as an add-on to conventional cancer drugs. It also has good antioxidant and anti-inflammatory properties, making it a promising candidate for anticancer therapy.

The RNA-seq experiments suggested that among the pathways affected by the GCDs, ferroptosis could be one of the pathways that could exert the GCD effects in cancer cells. Ferroptosis results from iron-dependent lipid peroxide accumulation (37). During iron-involving oxidative phosphorylation in mitochondria, the cells produce ROS and adenosine triphosphate (ATP) (37). Excess ROS can cause detrimental effects on the cells, and cancer cells can be more sensitive to ROS because of dysfunctional repair mechanisms (38, 39). On the other hand, ROS can also activate survival mechanisms, and the balance between the death and survival mechanisms will determine the cells’ fate (38, 39). The cancer types and cell lines can also react differently to ROS (40). The present study showed that GCDs increased ROS in squamous carcinoma cell lines, but the results should be confirmed in other cancer types. Increasing ROS levels to kill tumor cells without significantly affecting normal cells has been previously shown (41). Ginsenosides contained in Ginseng are known to increase ROS production in cancer cells (40), and it is, therefore, possible to achieve a tumor cell-killing effect without significant damage to normal tissues, as observed in the present study.

In the present study, oxidative phosphorylation showed the highest change after GCD treatment, probably related to ROS expression and ferroptosis of the cancer cells. In the pathway analysis, the ferroptosis-related pathways were expected to change based on the RNA-seq analysis after GCD treatment. Indeed, the ferroptosis was expected to be associated with the death of the cancer cells, and detection of ferroptosis was carried out to confirm the hypothesis. The results of the GO analysis showed that the differentially expressed genes were significantly enriched in terms of membrane remodeling and hypoxia, and the KEGG analysis revealed a significant concentration in the HIF-1 pathway. Under low oxygen conditions, known as hypoxia, cells face the risk of cellular dysfunction and death. To counteract these effects, the cell activates two separate processes: the hypoxia-inducible factor-1 (HIF-1) pathway and membrane remodeling. The HIF-1 pathway regulates the expression of genes involved in cellular processes necessary for survival under hypoxia, such as angiogenesis, glycolysis, and erythropoiesis. There can be interconnections and interactions between the HIF-1 pathway and membrane remodeling in hypoxic environments. HIF-1 activation can change the expression of genes involved in membrane remodeling, and changes in the composition of cellular membranes can affect the localization and stability of HIF-1. Meanwhile, membrane remodeling refers to changes in the composition, structure, and function of cellular membranes in response to signals and stimuli, which can modulate cellular processes like ion transport, signaling, and cell adhesion under hypoxic conditions. HIF-1 has been shown to regulate the expression of some genes involved in ferroptosis, such as glutathione peroxidase 4 (GPx4). Additionally, the hypoxic environment that activates the HIF-1 pathway can also trigger ferroptosis, particularly under certain conditions of oxidative stress. Ferroptosis is a form of regulated cell death triggered by the accumulation of iron and lipid peroxides and has been implicated in diseases like neurodegeneration and cancer. There can be some interconnections and interactions between membrane remodeling and ferroptosis in certain cellular contexts. Changes in the lipid composition of cellular membranes during membrane remodeling can alter the susceptibility of cells to ferroptosis. These results suggest that GCDs might regulate cell ferroptosis (42). The present study showed that GPX-4 was decreased, and COX-2 was increased in Cal-27 incubated with GCDs at 300 μg/mL. GPX-4 is an enzyme that decomposes H_2_O_2_ and organic H_2_O_2_ into water or corresponding alcohols (43), and inhibition of GPX-4 leads to ferroptosis (37). COX-2, also known as prostaglandin-endoperoxide synthase 2, is a suitable marker for lipid peroxidation in ferroptosis (44). Ferroptosis is a ROS-dependent cell death, and ROS is the incentive factor (37, 44). The intracellular ROS assay showed higher ROS levels in GCD-treated cells than in controls.

One of the leading causes of cancer death is tumor metastasis and invasion, which are caused by an increase in tumor cell motility and invasiveness (8, 10, 11). In this way, it was expected that Cal-27 cells and SCC-7 cells treated with GCDs should have a decreased cellular migration, which was observed in the present study. Previous studies also support Ginseng’s effect on cancer cells (45–47).

Ferroptosis is involved in the tumor immune microenvironment, where it contributes to the release of damage-associated molecular patterns (DAMPs) or lipid metabolites that can activate immune cells or mediate phagocytosis to maintain immune activation (48). Many tumors are characterized by an immune tolerant microenvironment that prevents the killing of cancer cells by innate immunity (49, 50). The feature of immune tolerance (or immune escape) are complex but can involve a decreased presence of T cells in the tumor microenvironment (49, 50), even reaching a state of “immune desert” (51). The present study suggests that GCDs led to increased infiltration of CD4^+^ T cells in the tumor and a decreased presence of regulatory T cells, suggesting an increased immunity in the tumor microenvironment and consistent with the role of ferroptosis in the tumor microenvironment (48). These results are supported by a recent study that suggested that Ginseng could potentiate cancer immunotherapy (24). This effect of Ginseng is promising, considering the actual development of immunotherapy, and warrants further study. The present study suggests the hypothesis that this effect of Ginseng on tumor immunity could be mediated, at least in part, by increased ferroptosis.

This study has limitations. Only squamous carcinoma cell lines were tested. Other cancer types should also be tested. Even though the RNA-seq experiment revealed pathways that GCDs could influence, confirmation of the exact genes and proteins involved should be performed. In addition, mechanisms other than ferroptosis can be involved. Future studies should examine the effects of GCDs in cancer cells more in-depth.

In conclusion, GCDs were successfully synthesized using the solvothermal method. The resulting GCDs had good biocompatibility in normal cells but could kill cancer cells. This death involved ferroptosis. GCDs could also inhibit the migration and invasion of cancer cells. The *in vivo* experiment revealed that GCDs could decrease tumor growth and that ferroptosis was involved. GCDs should be widely studied for developing novel nanoparticles-based medicine for cancer treatment and provide new methods for pharmaceutical research.

## Data availability statement

The original contributions presented in the study are included in the article/**Supplementary Material**. Further inquiries can be directed to the corresponding authors.

## Ethics statement

All animal experiments complied with the ARRIVE guidelines. This study was carried out in accordance with the principles of the Basel Declaration and the National Research Council’s Guide for the Care and Use of Laboratory Animals. The protocol was approved by the ethic committee.

## Author contributions

JL and ZW carried out the studies, participated in collecting data, and drafted the manuscript. ZG, HW, YL, and WW performed the statistical analysis. JH and CZ participated in its design, analysis, and draft the manuscript. All authors contributed to the article and approved the submitted version.

## Supplementary material

Supplementary Figure 1 Histological examinations of heart, liver, spleen, and kidney in the control and ginseng-based carbon dots (GCDs)-treated mice.

Supplementary Figure 2 Infiltration of immune cells in the tumors of mice treated or not with ginseng-based carbon dots (GCDs). **(A)** CD4^+^ lymphocytes. **(B)** Regulatory T cells. **(C)** Quantification of A and B. Data are presented as mean ± SD from three experiments. * P<0.05.

Supplementary Figure 3 Live/dead cell staining of RCD treated Cal-27 cells, red fluorescence represents dead cells, green fluorescence represents live cells.

Supplementary Figure 4 Quantification of ROS expression of . Data are presented as mean ± SD from three experiments. * P<0.05.

Supplementary Figure 5 ROS expression by flow cytometry. **(A)** Images of ROS expression by flow cytometry in Cal-27 cells treated with concentrations of GCDs with 500 μg/mL. **(B)** Quantification of ROS expression. Data are presented as mean ± SD from three experiments. * P<0.05.

Supplementary Figure 6 RNA sequencing of cells treated with ginseng-based carbon dots (GCDs). **(A, B)** Bubble plot of GO and KEGG analysis for all gene rates with altered expressions in Cal-27 cells treated with GCDs.
