# Supplementary material for: Ginseng-based carbon dots inhibit the growth of squamous cancer cells by increasing ferroptosis
Source: Front Oncol. 2023 Mar 10;13:1097692. doi: 10.3389/fonc.2023.1097692 (PMC10036825; doi:10.3389/fonc.2023.1097692)
Supplement: Supplementary file 1 [file Table_1.docx]

**supplementary Materials**

**Synthesis and purification of GCDs**

The GCDs were synthesized using the solvothermal procedure (1). Concisely, dry ginseng was smashed into powder by a micro mill, and then 1.0 g of powder was added to 10 mL of water and stirred well. The solution was transferred to a poly (tetrafluoroethylene) Teflon-lined autoclave (20 mL) and heated in an oven at 200℃ for 8 h. The oven was cooled to room temperature. The resulting mixture was centrifuged (8000 r/min, 10 min), and the supernatant solution was collected. The dark-yellow solution obtained from the supernatant solution was filtered through a 0.22-µm polyethersulfone membrane to remove large particles. For further purification, the GCDs aqueous solution was dialyzed in deionized water for 24 h in a dialysis bag to get rid of unreacted reagents and other small by-products. Finally, the purified GCDs aqueous solution was condensed in a vacuum rotary evaporator, and the GCDs were re-dissolved in deionized water for future use.

**Characterization of the GCDs**

A Lambda 800 ultraviolet-visible (UV-vis) spectrophotometer (Perkin-Elmer Life Sciences, Waltham, MA, USA) was used for obtaining the UV-vis absorption spectra. Photoluminescence spectroscopy (PL) was performed using a Lambda 950 spectrophotometer (Perkin-Elmer Life Sciences). High-resolution transmission electron microscopy images were captured with a CCD camera using a JEM-2100F electron microscope (JEOL, Tokyo, Japan) at 200 kV acceleration voltage. Energy dispersive spectra were measured by an Inca X-Max instrument (Oxford Instruments, Abingdon, United Kingdom). The binding energy calibration was based on 284.6 eV of C 1 s. Confocal laser scanning microscopy (CLSM) images were provided with a Fluoview FV1000 microscope (Olympus, Tokyo, Japan). CCK-8 measurements were performed using a microplate reader RT-6000 (Rayto Life and Analytical Sciences Co., Shenzhen, China).

**Cell culture**

The HaCaT, Cal-27, SCC-25, and SCC-7 cell lines were cultured in DMEM with high glucose containing 10% FBS, 100 U mL-1 penicillin, and 100 µg mL-1 streptomycin in a 5% CO_2_ humidified incubator at 37°C. The medium was changed every 2-3 days.

**ROS detection**

Cells were seeded on confocal dishes and grown overnight. ROS levels were measured using DCPH-DA reagent (Beyotime, S0033S). Cells were incubated with DCPH-DA at a final concentration of 10 μM in FBS-free DMEM for 30 min at 37°C protected from light, washed three times with FBS-free DMEM and resuspended in ice-cold PBS for observation using confocal laser scanning ROS by microscope (Olympus, FV3000). The working concentration of the ferroptosis inhibitor liproxstatin-1 was 200 nM, and the cells were pretreated for 24 h. The ImageJ software is used to analyze fluorescence images. After extracting the fluorescence intensity and distribution information, different regions are marked on the image. The fluorescence intensity of each region is analyzed and the fluorescence intensity of two different samples is compared to obtain statistical data on ROS expression.

**CCK-8 assays**

The cells were plated in a 96-well plate with 1×10^4^ cells per well and cultured overnight. After removing the medium, the cells were treated with 200 µL of medium containing different concentrations of GCDs and cultured for 24 h. Then, 10 µL of CCK-8 was added to each well for another 4 h’s incubation. Finally, a microplate reader was used to measure the absorbance at a wavelength of 450 nm. The cell viability was presented as a percentage of the control group.

**Flow cytometry**

The cells were seeded in a 6-well plate at 2×10^5^ cells per well and cultured overnight. The medium was replaced with 2 mL of medium containing different concentrations of GCDs. After 24 h, the cells were collected and washed twice with chilled PBS and resuspended in 100 µL of binding buffer. An annexin V-FITC/PI apoptosis assay kit or ROS assay kit was used to evaluate cell viability, and analysis was conducted through fluorescence-activated cell sorting.

**Western blot**

The cells were seeded in 6-well plates and cultured overnight. Then, 2 mL of medium containing different concentrations of GCDs was used to replace the medium. After 24 h, the cells were lysed with RIPA buffer to obtain cellular proteins. The protein extracts were run on SDS-PAGE and transferred on polyvinylidene fluoride membranes. The blot was sealed with 5% BSA at room temperature for 1 h and incubated with primary antibodies overnight at 4°C. Afterward, secondary antibodies were added and incubated at room temperature for 1 h. Enhanced chemiluminescence reagent was used for detecting the signals, and spectral band density was analyzed using the ImageJ software.

**Transwell assay**

The migration ability was evaluated using 24-well Transwell chambers equipped with polycarbonate membranes (pore size of 8 µm in diameter). The cells were incubated with different concentrations of GCDs for 24 h. The cells were collected and seeded at 5×10^4^ cells per well in the upper chambers and cultured with DMEM. DMEM containing 10% FBS was added to the lower chambers. After 24 h, the cells on the inferior membrane were fixed with methanol and stained with crystal violet. Images of the cells were taken under a microscope.

**Wound healing assay**

The cells were seeded in 6-well plates with 2×10^5^ cells per well and cultured until complete confluence. Three parallel scratches were made in each well with the tip of a 200-µl pipette, and the scratch area was measured. The culture medium was taken, and 2 mL of FBS-free medium containing different concentrations of GCDs was added to each well. Then, 24 h later, the cells were fixed with methanol and stained with crystal violet. Images of the scratched areas were captured. The scratch areas were observed with a microscope and analyzed with the ImageJ software.

**Live/Dead staining**

Prepare a mixture of the Live/Dead staining solution including two dyes: one for staining live cells (calcein) and another for staining dead cells (Pi). Add the staining solution to the cell culture and let it incubate for 15-30 minutes. Wash the cells with PBS to remove any unbound staining agents. Observe the cells under a fluorescent microscope with filters to detect the fluorescence of both dyes. Live cells will appear green, while dead cells will exhibit a red fluorescence. Finally, capture images or quantify the percentage of live and dead cells.

**Animal experiments**

Animal experiments were performed as previously described (2). Male C57BL/6 mice aged 8 weeks were randomly divided into two groups, including control and GCDs (n=5/group). SCC-7 cells in the logarithmic growth phase were washed twice with pre-warmed PBS, trypsinized, centrifuged, and resuspended in an H-DMEM medium. Then, 2×10^5^ cells were injected into each mouse using an insulin needle. The volume of cell suspension was 100 uL. The mice were anesthetized with isoflurane, and the inoculation site was disinfected with 75% alcohol. After injection, the skin was slightly raised, and the infection and tumor formation of the tumor site in nude mice were observed every day. About 1 week later, obvious nodules were visible at the inoculation site. The length L (length) and width W (width) of the tumor were measured with vernier calipers every 3 days. The mice were weighed with a balance. Tumor volume was calculated according to the tumor volume formula: V (mm^3^) = 1/2 × L (length) × W (width)^2^. When the tumor volume reached 100 mm^3^, the mice were randomly divided into two groups of five to ensure that their initial tumor volume was approximately the same. The mice were given PBS or GCDs 5 μg/mL, 100 μL, once every three days. A small amount of tumor was taken and embedded in OCT. The rest of the tumor was fixed in 4% paraformaldehyde and used for immunohistochemistry staining. The heart, liver, spleen, and kidney were collected and fixed, followed by HE staining.

**Statistical analysis**

All experiments were repeated at least three times. All data were expressed as means ± standard deviations (SD). First, Levene’s tests were performed for homogeneity of variance, and all P-values were >0.05, indicating that the homogeneity of variance within each population was equal. One-way ANOVA and Bonferroni post hoc analysis was used to evaluate the statistical differences among the groups. P<0.05 indicated that the differences were statistically significant.

**REFERENCES**

1. Liu J, Geng Y, Li D, Yao H, Huo Z, Li Y, et al. Deep Red Emissive Carbonized Polymer Dots with Unprecedented Narrow Full Width at Half Maximum. *Adv Mater* (2021) 33(38):e2007162. Epub 2021/09/22. doi: 10.1002/adma.202007162.

2. Wang D, Xu X, Zhang K, Sun B, Wang L, Meng L, et al. Codelivery of Doxorubicin and Mdr1-Sirna by Mesoporous Silica Nanoparticles-Polymerpolyethylenimine to Improve Oral Squamous Carcinoma Treatment. *Int J Nanomedicine* (2018) 13:187-98. Epub 2018/01/19. doi: 10.2147/IJN.S150610.
