# Supplementary figures and images for: Ginseng-based carbon dots inhibit the growth of squamous cancer cells by increasing ferroptosis
Source: Front Oncol. 2023 Mar 10;13:1097692. doi: 10.3389/fonc.2023.1097692 (PMC10036825; doi:10.3389/fonc.2023.1097692)

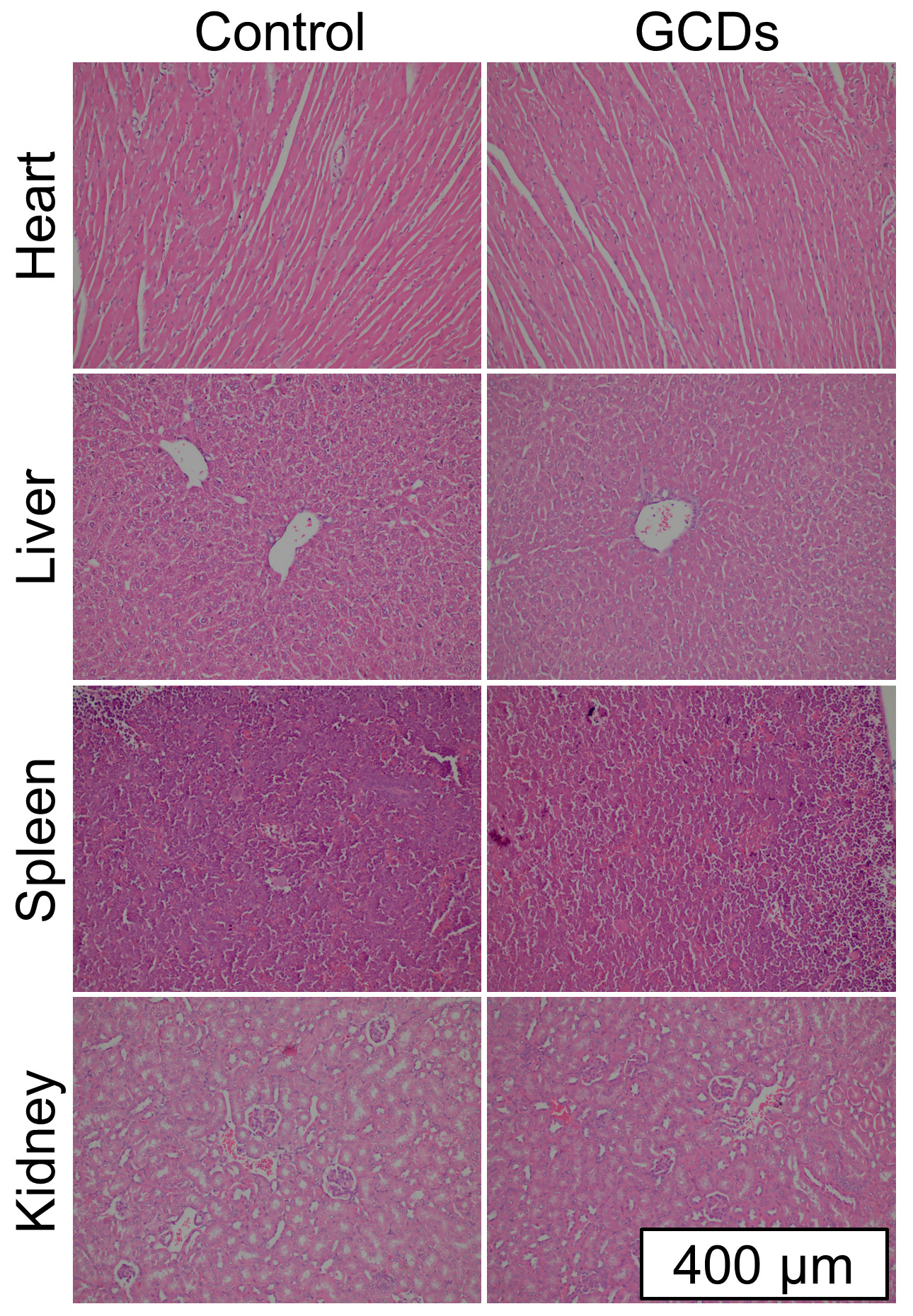

Supplement: Supplementary Figure 1 — Histological examinations of heart, liver, spleen, and kidney in the control and ginseng-based carbon dots (GCDs)-treated mice. [file Image_1.jpeg]

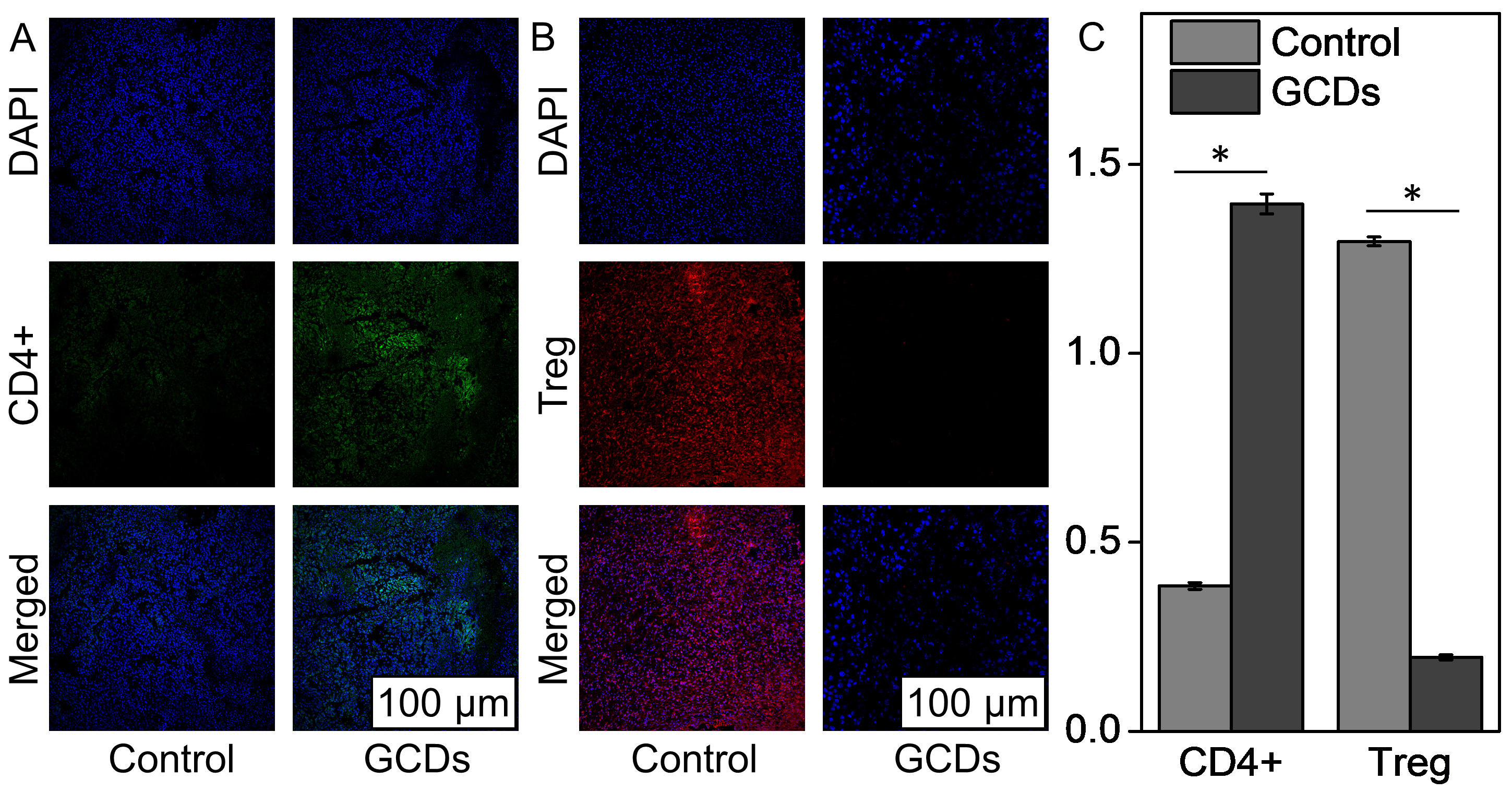

Supplement: Supplementary Figure 2 — Infiltration of immune cells in the tumors of mice treated or not with ginseng-based carbon dots (GCDs). (A) CD4+ lymphocytes. (B) Regulatory T cells. (C) Quantification of A and B. Data are presented as mean ± SD from three experiments. * P<0.05. [file Image_2.jpeg]

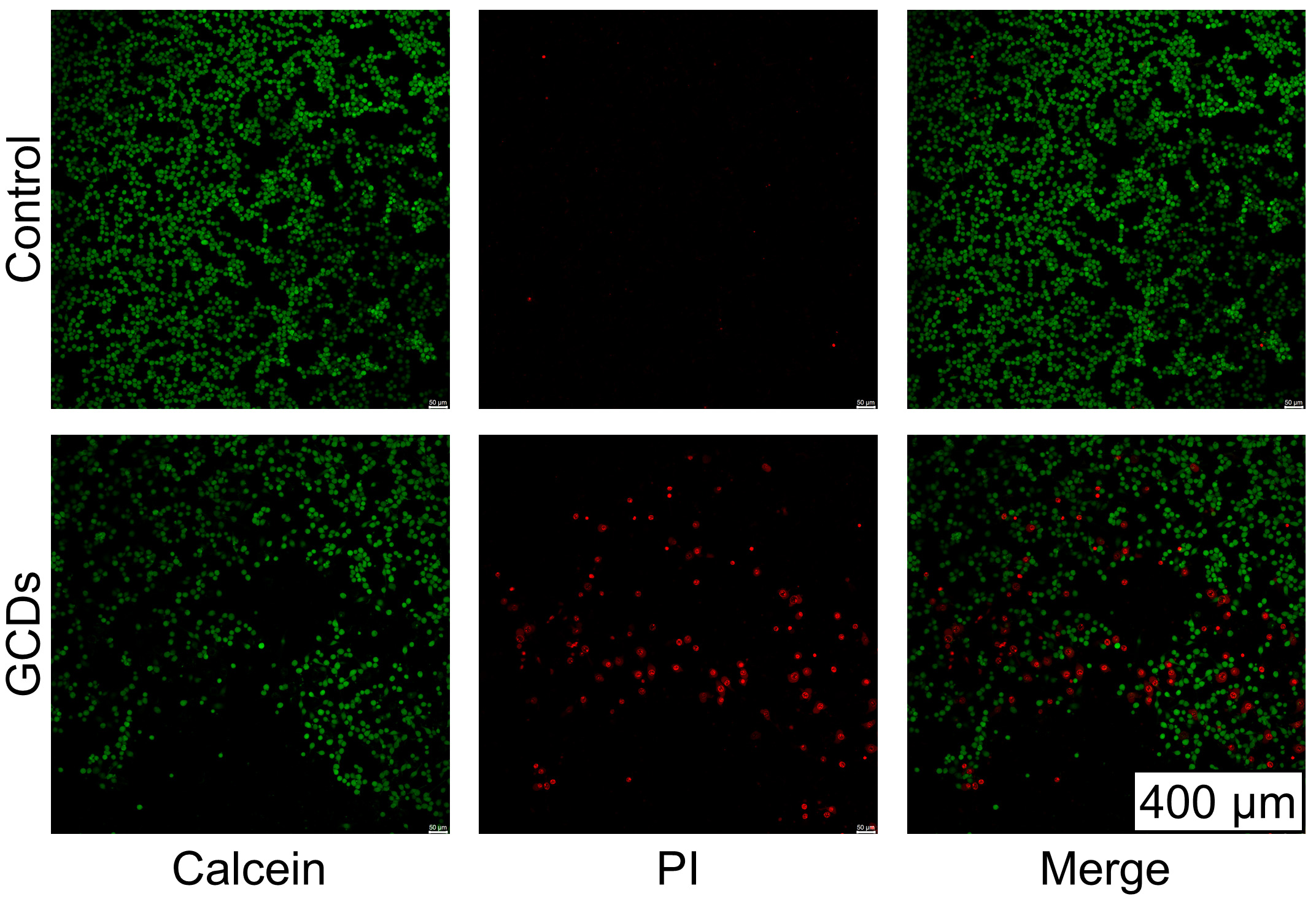

Supplement: Supplementary Figure 3 — Live/dead cell staining of RCD treated Cal-27 cells, red fluorescence represents dead cells, green fluorescence represents live cells. [file Image_3.jpeg]

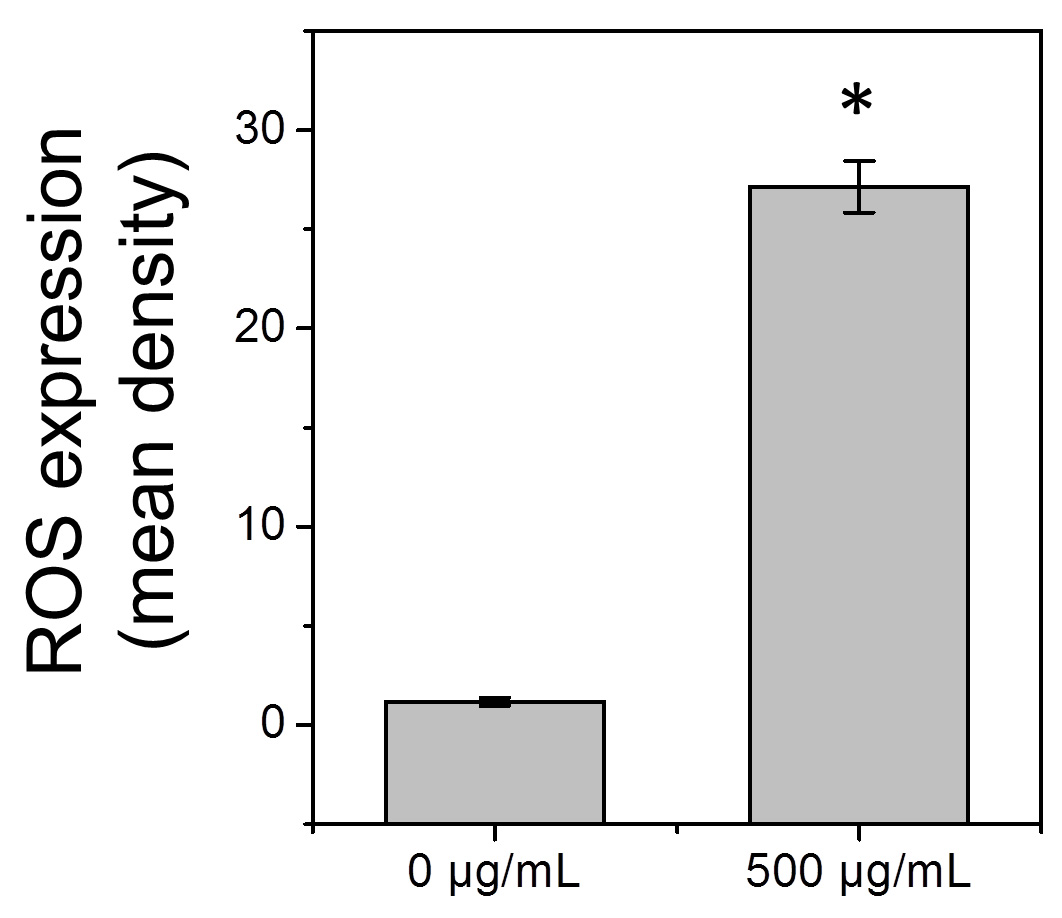

Supplement: Supplementary Figure 4 — Quantification of ROS expression of . Data are presented as mean ± SD from three experiments. * P<0.05. [file Image_4.jpeg]

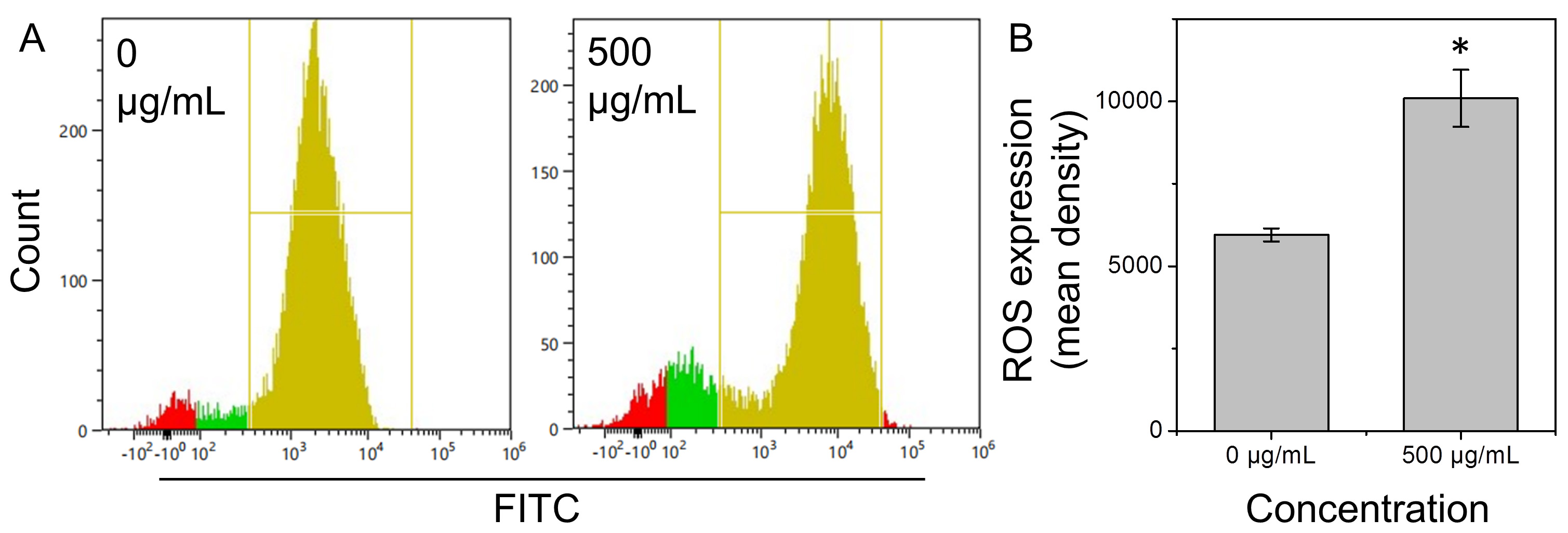

Supplement: Supplementary Figure 5 — ROS expression by flow cytometry. (A) Images of ROS expression by flow cytometry in Cal-27 cells treated with concentrations of GCDs with 500 μg/mL. (B) Quantification of ROS expression. Data are presented as mean ± SD from three experiments. * P<0.05. [file Image_5.jpeg]

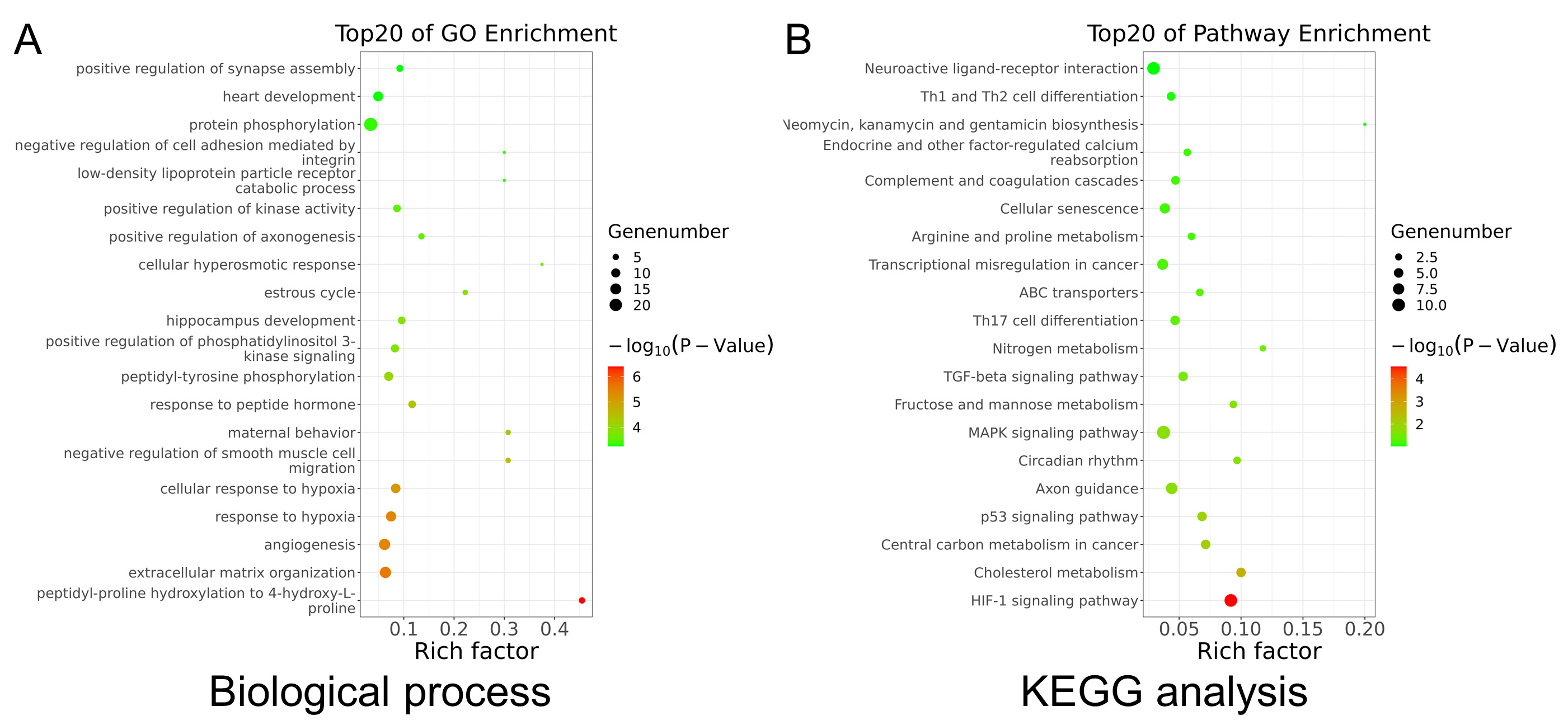

Supplement: Supplementary Figure 6 — RNA sequencing of cells treated with ginseng-based carbon dots (GCDs). (A, B) Bubble plot of GO and KEGG analysis for all gene rates with altered expressions in Cal-27 cells treated with GCDs. [file Image_6.jpeg]
